# Abstracts DGTHG

**DOI:** 10.1515/iss-2019-2009

**Published:** 2019-03-20

## DGTHG: Aortic surgery

### Short and long-term outcomes of open repair and fenestrated endografting of pararenal aortic aneurysms in a concurrent propensity adjusted comparison.

(Abstract ID: 471)

B. Fiorucci^1^, F. Speziale^2^, T. Kölbel^3^, N. Tsilimparis^1^, P. Sirignano^2^, L. Capoccia^2^, G. Simonte^4^, F. Verzini^4^

^1^*Uniklinik München*

^2^*Umberto I Hospital, Sapienza University of Rome*

^3^*Universitätsklinikum Hamburg-Eppendorf*

^4^*Santa Maria della Misericordia University Hospital, Perugia*

**Background:**

The best treatment of para-renal aortic aneurysms (p-AAA) is under investigation. Open surgical repair (OSR) is the gold standard but is associated to high surgical stress. Fenestrated endografts (fEVAR) require dedicated materials and specific expertise. Different characteristics of populations treated with fEVAR and OSR might influence short- and long-term results. Aim of the present study is to compare short- and long-term outcomes of patients treated with fEVAR and OSR for pararenal aortic aneurysms in an experience of high volume centers.

**Materials and methods:**

A multicenter retrospective analysis of p-AAA electively treated with OSR or fEVAR from 1998 to 2015 was performed at three tertiary institutions. Endpoints were 30-day morbidity and mortality, late reinterventions, visceral arteries occlusion and mortality. Analysis was conducted on entire population and on a propensity score-matched population. Propensity score was constructed on age, gender, coronary artery disease (CAD) and chronic renal failure.

**Results:**

200 patients (108 OSR, 92 fEVAR) were included. Mean age was 73±7 years. FEVAR patients were significantly older than OSR patients (p<.0005) and had more frequently CAD (p<.0005) and previous stroke (p=.003). OSR-patients had higher risk of perioperative morbidity (OR2.5, CI95% 1.09-5.71;p=.03), specifically respiratory failure (OR4.06, CI95% 1.12- 4.72;p=.034). These findings were confirmed in the propensity-adjusted analysis, where also cardiac complications resulted higher after OSR (OR12.8, CI95% 0.07-0.21;p=.02). No difference in peri-operative mortality was confirmed. Mean follow-up was 50 months (0-119). 5-years results showed higher survival and lower reintervention rates after OSR in the unmatched population, with a small but significant difference in the risk of late visceral artery occlusion-stenosis after fEVAR. At propensity analysis, no differences in late survival were found between groups.

**Conclusion:**

fEVAR and OSR may afford similar early and 5-year survival rates. Higher risks of peri-operative systemic complications after OSR are counter-balanced by higher risks of late visceral vessel patency issues and need of reintervention after fEVAR. Both procedures are safe and effective in the long-term in experienced centers, where patient evaluation should drive the treatment strategy.

### Impact of deep hypothermic circulatory arrest on liver function in patients undergoing surgical replacement of ascending aorta due to Aneurysm and calcification

(Abstract ID: 678)

M. Salem^1^, M. A. Salem^1^, Y. Erdal^1^, C. Friedrich^1^, K. Huenges^1^, B. Panholzer^1^, T. Pühler^1^, J. Schoettler^1^, F. Schoeneich^1^, J. Cremer^1^, A. Haneya^1^

^1^*Universitätsklinikum Schleswig-Holstein, Kiel*

**Background:**

The effect of deep hypothermic circulatory arrest on different body organs is still not well investigated enough. Failure of liver function after surgical replacement of ascending aorta in DHCA in patients due to aneurysm or calcification represents nowadays a major concern. A failing liver cannot produce enough clotting factors, leading to extensive bleeding. This major study focused on the impact of DHCA on hepatic function in those patients.

**Materials and methods:**

The study analyzed 905 consecutive operation between 2001 and 2015 retrospectively, included (male 66.7% vs. female 33.3%) undergoing replacement of ascending aorta using DHCA due to aneurysm or calcification. All Cases with type A-dissection of ascending aorta are excluded from the study. Bilirubin, GOT and GPT as parameters for liver function were documented pre- and postoperatively till 8 days. The potential correlation of the length of DHCA and worsening of liver function was evaluated using Spearman’s rank correlation.

**Results:**

The mean age was 66.7 /-11.1years and 33.3% of the patients were female. Intraoperative data revealed a median aortic cross clamp time of 92min (65;125). The median duration of DHCA was 14min (12;18). The 30 day mortality was 4% (n=36). The study showed no significant correlation between the length of DHCA and the postoperative liver function after surgery until postoperative day 8, (Spearman’s correlation coefficient). The multivariate logistic regression analysis suggests that age (OR 2.799; p<0.019) was an independent risk factor for mortality as well as cross clamping time (OR 0.985; p<0.033) and bypass time (OR 1.022; p<0.001).

**Conclusion:**

In our analysis, there is no correlation determined between the deep hypothermic circulatory arrest and postoperative worsening of liver function.

## DGTHG: Cardiac electrical implantable devices

### Pacemaker, ICD, CRT implantation: A retrospective analysis from 2002 to 2018 in the Department of Cardiac Surgery BwZK

(Abstract ID: 542)

A. Alhumidi^1^, A. Alsoliman^1^, E. Szilagyi^1^, R. Feyrer^1^

^1^*Bundeswehrzentralkrankenhaus Koblenz*

**Background:**

Background: At the Central Military Hospital in Koblenz all cardiac electrical interventions have been performed by the Department of Cardiac Surgery for more than 20 years. In this study, a retrospective analysis of nearly 4000 patients with focus on outcome as well as peri- and postoperative complications was performed.

**Materials and methods:**

Material and Methods: In the period from 2002 to 2018, a total of 3942 cardiac electrosurgical procedures were performed: 81% as elective, 10% as urgent and 95% as emergency intervention. 62.1% of the patients had pacemaker surgery, 30.8% ICD surgery and 6.2% CRT surgery. By age group, 25% of patients were between 60 and 70 years, 37% between 70 and 80 years and 15% between 80 and 90 years, 72% of them men and 28% women. The procedures were performed in 81% of the cases under local anesthesia.

**Results:**

Results: The perioperative / postoperative complications included hematomas in 0.76%, pneumothorax in 0.31%, wound healing disorders 0.1%, pericardial tamponade with 0.12% of cases, mortality 0.076%. The functional success rate was 98.2%.

**Conclusion:**

Discussion: The results of the follow-up show that cardiac electrical interventions can be performed in the hands of cardiac surgeons with great certainty and excellent success rate and low complication rate. The complication rates are below the numbers described in the literature, occurring complications can be treated on the spot.

### Surgical management of complications related to transvenous lead extraction

(Abstract ID: 626)

T. Madej^1^, M. Knaut^1^, K. Matschke^1^

^1^*Universitätsklinikum Dresden*

**Background:**

Despite increasing number of cardiac implantable cardiovascular device (CIED) lead extractions, management of extraction-related complications was not yet sufficiently explored. The aim of this study is to describe the incidence and management of periprocedural complications during transvenous lead extraction (TLE)

**Materials and methods:**

We retrospectively reviewed the database of all patients undergoing TLE at our institution during the period 09/2011 - 07/2018. Procedural characteristics and related complications were evaluated according to severity (major, minor) and their treatment was analysed.

**Results:**

A total of 670 leads were extracted in 284 consecutive patients (635 leads complete, 24 leads partial, 11 leads failed extraction). Indications for TLE were local infection in 173 (61%), systemic infection in 62 (22%) and lead dysfunction and/or venous occlusion in 49 patients (19%). Minor complications included pocket bleeding requiring re-exploration (9 patients, 3.2%) and pneumothorax requiring chest tube (2 patients, 0.7%). Major complications (4 cases, 1.4%) included 3 vascular and 1 cardiac tears. All these injuries led to rapid circulatory decompensation and were attempted for repair via immediate median sternotomy. In the first case (pocket infection), bleeding from innominate vein/superior vena cava (SVC) could not be stopped because of severe adhesions after previous cardiac surgery, and a massive bleeding to the right pleura resulted in intraoperative death. In the second case (lead endocarditis), bleeding from SVC below pericardial reflection was repaired with patch, but the patient died after weaning from cardiopulmonary bypass (CPB) due to uncontrollable sepsis resulting from preoperative systemic infection. Third and fourth patient (SVC occlusion and lead dysfunction) survived SVC tear and RV apex tear which could be directly sutured without CPB. Both survivors recovered ad were discharged 7 and 10 days after surgery. Overall intraoperative mortality was 0.7%. Both deaths (and all 3 SVC tears) occurred before the introduction of "bridge occlusion balloon" (BOB).

**Conclusion:**

In this analysis, major and minor TLE-related complications occurred in 1.4 and 3.9%, respectively. Immediate surgical response enabled repair and complete recovery in 2 of 4 patients with severe vascular/cardiac tear. The role of BOB remains unclear and its effectivity should be addressed in a prospective study.

### Vacuum assisted wound therapy in a case of severe pocket hematoma avoids explantation of a cardiac resynchronization device (CRTD) in a high risk octogenarian patient

(Abstract ID: 657)

A. Thiem^1^, M. Salem^1^, T. Pühler^1^, A. Haneya^1^, J. Cremer^1^

^1^*Universitätsklinikum Schleswig-Holstein, Kiel*

**Background:**

Pocket hematoma following implantation of a cardiac resynchronization device (CRTD) is a severe complication and may lead to device infection and endocarditis. Consequent surgical treatment such as device removal and lead extraction is sometimes complex and at high risk. We hereby report a case of a multimorbid octogenarian suffering from a giant pocket hematoma following CRTD exchange and additional implantation of a right ventricular chamber lead under anticoagulation with a factor Xa inhibitor (Apixaban®). Instead of removing the over 4 years old leads revision of the hematoma and additionally temporary vacuum assisted closure (V.A.C.) was performed.

**Materials and methods:**

A 82 year old female patient, already supported with a two-chamber CRTD, underwent a pacemaker re-operation as a second implant, adding a dual-coil shock lead to the right ventricle chamber, due to a low battery state of the device and lead dysfunction.With a history of cerebral embolism, atrial fibrillation, history of complex cardiac surgery, including myocardial revascularization and mitral valve repair with poor left ventricular function the patient was under anticoagulation with Aspirin® and Apixaban®. Though the anticoagulation regime was paused perioperatively, a slight swelling at the pacemaker implant site was revealed. Additionally a few days later the patient experienced dysarthria and dizziness and was transferred to the in house stroke unit. Oral anticoagulation was recommenced immediately, suspecting a further cerebrovascular event. Three weeks later, after discharge, the patient presented with a painful giant hematoma on the left pocket site. The swelled cutis was nearly perforated. Because of acute pain an urgent operative revision was performed. After removal of the hematoma, extensive surgical debridement and hemostasis was conducted curefully. The three leads and the device were disinfected and the wound was temporary closed by V.A.C. therapy. As an alternative treatment a laser-guided lead extraction or open-chest-re-do surgery for lead removal would have been mandatory.

**Results:**

The wound treated by negative pressure showed recompensation at a good pace. Surgical cleaning inclusive debridement and moderate necrosectomy was performed every 4 days. After changing V.A.C. over 3 weeks the wound could be closed surgically in good conditions leaving the leads and device in the pocket. Although all microbiological findings were negative, antimicrobial therapy was prophylactically administered for 10 days. No recurrent wound and no lead- or device infection occurred so far. In this case V.A.C. therapy led to a satisfactory wound recovery with an appropriate state for surgical wound closure. High risk removal re-do-surgery was successfully avoided.

**Conclusion:**

V.A.C. treatment may be an option for selected patients with pacemaker site complications for whom the risk for a laser-guided lead extraction or surgical removal of the device and electrodes as primary therapy is too high. Furthermore, also devastating wound conditions could be turned into complete recovery allowing to leave leads and device in situ with satisfactory midterm outcome.

### Epicardial implantation of cardioverter/defibrillator in children and adults with congenital heart disease

(Abstract ID: 852)

T. Tirilomis^1^, M. Großmann^1^, M. Friedrich^1^, C. Bireta^1^, W. Ruschewski^1^, D. Zenker^1^

^1^*Universitätsmedizin Göttingen*

**Background:**

Congenital heart diseases may develop significant arrhythmias resulting in implantation of a cardioverter-defibrillator device (ICD). Because of the body size of pediatric patients and the underlying anatomy after cardiac surgery very often transvenous implantation is not possible resulting in use of epicardial or combined implantation techniques. The aim of the study was the analysis of indications and outcome of epicardial ICD procedures.

**Materials and methods:**

Medical records of children and adults with congenital heart disease who underwent defibrillator implantation were reviewed. From 2004 to 2015 epicardial defibrillator implantation was performed in 33 patients. Twenty patients were male and 13 female. Median age was 6 years and ranged from 5 months to 50 years.

**Results:**

Twenty-seven patients (82%) received a 1-chamber device and the remaining six patients (18%) a 2-chamber device. A sympathectomy was simultaneously performed in four patients (12%) and another two patients (6%) underwent defibrillator implantation along with open-heart surgery.

Five patients (15%) had undergone second procedure with exchange of defibrillator electrodes (n=2; 2 years and 3 years after initial procedure respectively), placement of an additional subpleural/subcutan coil (n=2; 2 days and 1 year after initial procedure), or exchange of subpleural coil (n=1; 9 years after initial procedure).

**Conclusion:**

Epicardial ICD surgery in children and adults with congenital heart disease is challenging and increasingly complex. Careful follow-up is mandatory.

## DGTHG: Modern surgical options in aortic and thoracic surgery

### Percutaneous stereotactic CT-guided microwave ablation for colorectal lung metastases using high-frequency jet ventilation

(Abstract ID: 109)

S. Salm^1^, M. Maurer^1^, S. Sesia^1^, T. Stoker^1^, D. Candinas^1^, A. Lachenmayer^1^

^1^*University Hospital Bern*

**Background:**

Minimally invasive and tissue-sparing tumor destruction techniques play an increasingly important role in oncologic patients. Thermal ablation is routinely used for the treatment of malignant liver lesions and has recently been introduced for patients with lung metastases, in particular those with reduced lung reserve or multiple lung metastases requiring multiple treatments during the course of their disease. Stereotactic image-guidance and computer-assisted navigation technology can be used to increase the precision of needle placement and allows a safe treatment of small lesions in difficult to resect anatomic regions. With experience in treating more than 400 liver lesions with this technique, we aimed to present this first report of stereotactic CT-guided microwave ablation for colorectal lung metastases.

**Materials and methods:**

Retrospective data analysis of 2 patients treated for colorectal lung metastases at the Department of Visceral Surgery and Medicine, the Department of Thoracic Surgery and the Department of Radiology of the University Hospital Bern, Switzerland. All procedures were performed under general anesthesia in the interventional CT suite by an interdisciplinary team consisting of specifically trained surgeons and interventional radiologists. High Frequency Jet ventilation was used to ensure minimal movement of the patient and the diaphragm during the procedure. A commercially available navigation system (CAS-ONE, CAScination AG, Bern, Switzerland) was used for trajectory planning (Fig. 1c), positioning of ablation probes and immediate treatment validation. Thermal ablation was performed using microwave energy (Acculis MTA System, AngioDynamics, Latham, NY, USA). The intervention always consisted of four main procedural steps: (1) planning, (2) navigation, (3) probe validation and ablation, (4) ablation zone validation.

**Results:**

We herein report the results of 2 patients who received a percutaneous stereotactic image-guided microwave ablation of colorectal lung metastases using high-frequency jet ventilation. Patient 1 was a 76-year-old man with an 8 mm metastasis in segment V/VI right (Fig 1). The ablation was performed for 4 minutes at 60 W. Patient 2 was a 71-year old woman with a 5 mm metastasis in the left lung, segment VI. The ablation was performed for 5 minutes at 35 W and additional 5 minutes at 65 W due to incomplete ablation after the first treatment. Lateral and longitudinal error of the needle placements were below 2 mm. Both patients had previous surgeries for colon cancer, liver and lung metastases. In both cases, the post-interventional CT scan showed a complete ablation of the metastasis (Fig 1b). Both patients were hospitalized for 1 and 2 days, respectively. No intra- or postinterventional complications occurred and none of the 2 patients required a thorax drainage after treatment. Radiological follow-up 6 months after the ablation showed no signs of local recurrence or tumor progression in patient 1, the other patient is currently still awaiting the follow-up.

**Conclusion:**

Percutaneous stereotactic CT-guided microwave ablation seems to be safe and feasible for the treatment of colorectal lung metastases. In the setting of personalized medicine with an increasing need for minimal-invasive and tissue-sparing tumor treatments, this technology might offer an interesting alternative especially for patients with multiple co-morbidities and those requiring multiple treatments. Clearly, further studies need to validate its safety and efficiency in the clinical setting.

**Picture:**
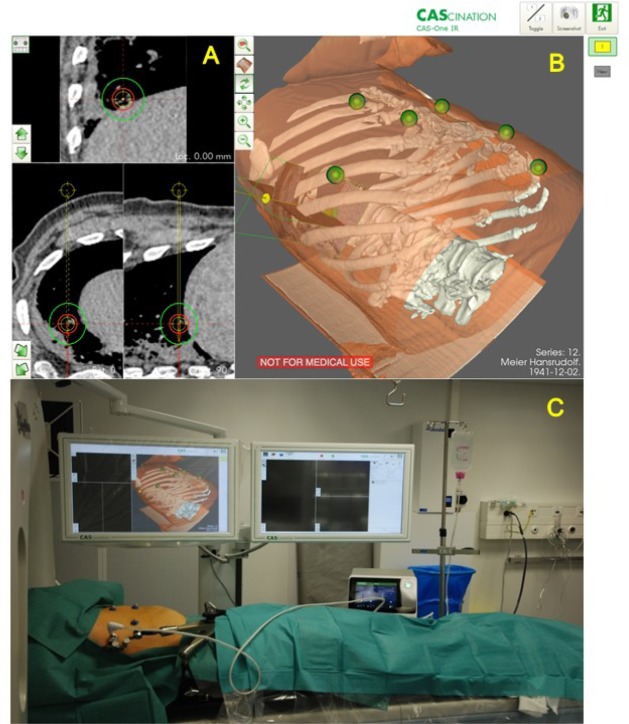
A) Planning of the ablation in the CT scan with targeting of the lung metastasis (red circle) and microwave ablation zone (green circle) B) Planning with the 3D-reconstruction to plan the pathway (yellow) and length of the needle and the six synchronization markers on the patient (green) C) Set-up with the two touch-screen

### The multi-modal treatment of a persistent lymphangiomatosis-induced chylothorax

(Abstract ID: 353)

B. A. Högerle^1^, H. Zabeck^1^, K. Storz^1^, M. Kreuter^1^, J. Debus^2^, S. Rieken^2^, H. Winter^1^

^1^*Thoraxklinik am Universitätsklinikum Heidelberg*

^2^*Universitätsklinikum Heidelberg*

**Background:**

Chylothorax is defined as the collection of chylous effusion in the pleural cavity. Lymphangiomatosis is a rare cause of a persistent chylothorax. Multiple treatment options are known to be effective. Most patients benefit from supportive measures and low-fat diet. If applicable, surgical resection and ligation of the thoracic duct can stop the chylous effusion. If the treatment is refractory, the management will be complex. The treatment must be chosen carefully as co-morbidities often lead to limitations.

**Materials and methods:**

In October 2016, a 73-years old femal patient was admitted presenting a recurrent right-sided chylothorax and several co-morbidities such as pneumonia, peripheral pulmonary embolism, phlebothrombosis, and chronic renal insufficiency. As it was persistent, a redo video assisted thoracic surgery (VATS) and a talc pleurodesis were performed, but with no effect. A computed tomography (CT) showed signs of a a new lymphangiomatosis-suspicious tumor of the left mediastinum. A left-sided thoracotomy for tumor removal and ligation of the thoracic duct was performed. Postoperatively, the pleural effusion was still persistent (right: up to 3 L/day; left: up to 200 mL/day) and could not be managed dietetically (oral or parenteral). Sirolimus was applied, but the treatment had to be interrupted due to side effects. A conventional lymphography was performed, but a definite leakage was not detected. As all options including diet, medication, and surgery were excluded and the clinical status of the patient deteriorated, we presented the case to our colleagues from the Department of Radiooncology and Radiotherapy. A low-dose radiotherapy was planned. The clinical target volume (CTV) included the thoracic duct, the juxtarenal, and the left supraclavicular lymph nodes, with a pre- and paravertebral safety margin of 5 mm. The total photon dose was 12 Gy, delivered in 12 fractions. After completing radiotherapy, the pleural effusion stopped almost instantly and permanently. The application of sirolimus was re-implemented, this time with better tolerability.

**Results:**

The patient recovered well and was discharged home on overall hospitalization day 139. She still takes sirolimus (1.5 mg/day). After a 18-month follow-up, there is still no evidence of a recurrent pleural effusion.

**Conclusion:**

Radiotherapy is an underestimated treatment option for lymphangiomatosis-induced chylothorax. The radiation-related risk of acute and chronic complications must be carefully considered. As relevant acute complications esophagitis and pneumonitis can occur due to the close vicinity. Secondary malignant tumors have been observed as further relevant chronic complications. The risk of both acute and chronic complications is assumed to be acceptable in the absence of alternatives.In conclusion, the management of a persistent lymphangioma-induced chylothorax is complex in case of resistance to standard treatment options and multi-morbid patients. A multi-modal treatment is especially suitable for older patients who cannot undergo surgery, do not initially respond to sirolimus, or suffer from other comorbidities which require an urgent end of the chylous effusion.

**Picture:**
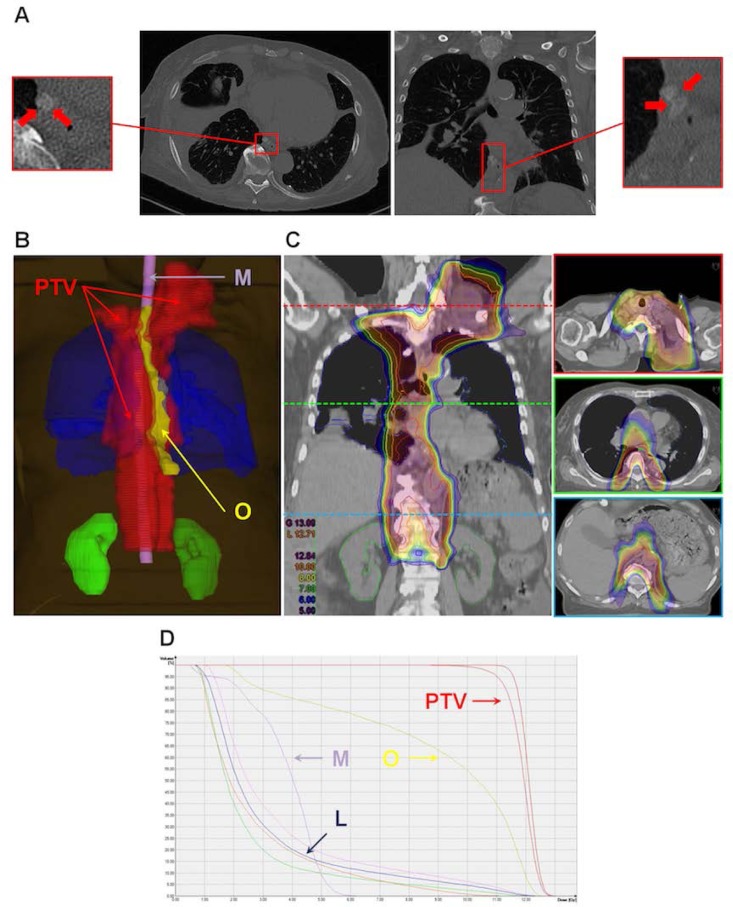
Lymphleckage, Planungsvolumen, Isodosenkurve

### Multidisciplinary Management in non-intubated uniportal VATS-Lobectomy

(Abstract ID: 560)

H. Starke^1^, N. Zinne^1^, F. Logemann^1^, A. Haverich^1^, P. Zardo^1^

^1^*Medizinische Hochschule Hannover*

**Background:**

Since being first reported by Giancarlo Roviario in the early 1990ies, minimally-invasive anatomical lung resection has gained widespread acceptance both nationally and internationally, and is increasingly considered gold standard for treatment of early-stage lung cancer. Even though most centers still prefer a so-called "triportal technique" while performing VATS-lobectomies, selected units favor Diego Gonzales Rivas´ uniportal approach, in which the 2 caudally placed incisions are omitted. Reported advantages of this technique include a further reduced operative trauma, less postoperative pain, better cosmetics whilst maintaining excellent oncological resection quality. Additionally, it is possible to altogether forego orotracheal intubation in highly selected patients with reduced pulmonary function to perform an anatomical lung resection under spontaneous breathing. This approach obviates classical drawbacks of mechanical ventilation as secretion of pro-inflammatory cytokines, induction of mechanical damage on alveolar level by barotrauma and atelectasis of the dependent lung after muscle relaxation. We would like to report our initial multidisciplinary team experience in managing these highly complex patients.

**Materials and methods:**

Prospectively collected data was retrospectively evaluated. We included all patients that underwent non-intubated uniportal major lung resection (niVATS) at our department between July and September 2018 after written informed consent. Our protocol included preoperative inhalatory local anesthetics, mild sedation with dexmedetomidine, either peridural analgesia or paravertebral blockade as well as vagal nerve blockade during the procedure. If required propofol was applied for deeper sedation, a laryngeal mask was inserted and anesthesia depth was monitored by EEG.

**Results:**

N=14 (8 males, age 69 +/-9 years) underwent niVATS during the monitored timeframe, and all patients adhered to our protocol. Mean procedure duration was 110 +/-50 minutes and 71% of cases were done in deeper sedation with laryngeal mask. During spontaneous ventilation maximal end tidal CO2 was 54±8 mmHg and lowest peripheral oxygen saturation was 93±4%. We had n=1 emergent conversion to conventional intubation due to technical reasons (centrally located tumour with complex bronchioplastic reconstruction), but all patients were transferred to our recovery room in hemodynamically and respiratory stable conditions. During this phase 57% of all patients required oxygen supply, which was discontinued after transferal to the ward. In the first 1-2 postoperative hours pain severity was assessed by numeric rating scale, and reported levels ranged from 0-3.

**Conclusion:**

Uniportal niVATS appears to be reasonably safe and associated with low postoperative pain scores and a quick postprocedural recovery. It needs to be stated that avoidance of orotracheal intubation may lead to uncontrolled patient movement, coughing, mediastinal shift or marked diaphragmatic excursion, thus being extremely challenging for surgeons and anesthetists alike, especially when emergent conversion to intubation and thoracotomy is required. Therefore, we recommend that this approach should be restricted to specialized surgeons and anesthetists well trained in minimally-invasive surgery.

### Three-Dimensional Aortic Model to Create a Fenestrated Stentgraft for the Urgent Treatment of a Para-visceral Penetrating Aortic Ulcer

(Abstract ID: 571)

D. Branzan^1^, D. Winkler^1^, A. Schmidt^1^, D. Scheinert^1^, I. Gockel^1^, R. Grunert^1^

^1^*Universitätsklinikum Leipzig*

**Background:**

To describe the role of three dimensional (3-D) printing in manufacturing a four vessel fenestrated physician modified stentgraft (PMSG) for the urgent treatment of a symptomatic para-visceral penetrating aortic ulcer (PAU).

**Materials and methods:**

A 61-year-old male patient with symptomatic PAU at the level of the superior mesenteric artery underwent an urgent treatment using a PMSG with four fenestrations. To reduce measurements errors and increase the accuracy of the localization of the openings in the stentgraft, a biocompatible 3-D hollow model of the aorta was created based on the computed tomography angiography (CTA) of the patient.

**Results:**

The stentgraft was deployed in the sterilized aortic model and the origins of the visceral vessels were marked accordingly. After cutting the fenestrations and adding radio-opaque markers around them, the stentgraft was re-sheathed and successfully deployed in the thoraco-abdominal aorta.CTA at discharge showed complete thrombosis of the PAU and patency of all visceral vessels.

**Conclusion:**

A 3-D sterilized model of the thoraco-abdominal aorta facilitates the on-table creation of fenestrations of the PMSG for the urgent treatment of thoraco-abdominal aortic pathologies.

## DGTHG: Management of wound infections

### Severe Complication of a Mediastinal Teratoma: Acute Pancreatitis in the Chest. A case report.

(Abstract ID: 115)

S. Taha-Mehlitz^1^, R. Schläpfer^1^, A. Leiser^1^

^1^*Luzerner Kantonsspital, Luzern*

**Background:**

Teratomas are the second most common lesions in anterior mediastinum besides thymoma, lymphoma, intrathoracic thyroid and bronchiogenic cysts. They usually occur in young adults with equal gender distribution. Alpha fetoprotein (AFP) and beta human gonadotropin (b-HCG) serum levels are not elevated in teratomas, opposite to germ cell tumors. Micro- and macroscopically teratomas exhibit a variety of tissues of all three germinal layers, that still fascinates us. We present a case of a young woman with teratoma including pancreatic tissue in the chest.

**Materials and methods:**

A 28-year-old woman presented with progressive chest pain, fever and dyspnea. She had tachypnea, tachycardia and right-sided attenuated breathing sounds. Along with leucocytosis and elevated C-reactive proteine, the chest X-ray showed a large pleural effusion on the right side. The CT scan revealed a partially locular and cystic mass of 13 cm in diameter in the anterior mediastinum leading to compression of the middle lobe. AFP and b-HCG levels were normal.

**Results:**

A chest tube on the right side was inserted and open biopsy of the tumor showed inflammatory myofibroblastic tissue. Because of distinctive adhesions and inflammation, tumor resection was performed via sternotomy and right anterior thoracotomy. An inflammatory cavity led to partial resection of the right middle lobe. The postoperative course was uneventful. Histopathology showed a benign teratoma including pancreatic glandular tissue. Five years after surgery, there is no recurrence and the patient is free of complaints.

**Conclusion:**

Pancreatic tissue is a typical component of mediastinal teratomas, what is not observed in gonadal teratomas. Besides asymptomatic incidental findings, mass effects cause symptoms like chest pain, dyspnea and cough. The pancreatic exocrine proteolytic enzymes may lead to perforation of the tumor into pulmonal parenchyma, pleural cavity with effusions as described in our case, empyema or cardiac tamponade. Erroding into the bronchiatic system, they may cause symptoms as coughing hair or sebum. Literature describes that teratomas with pancreatic glandular activity often cause adhesions and erosions in surrounding tissues due to inflammation. This may cause complicated surgical management. Complete surgical resection is mandatory to prevent from recurrence.

**Picture:**
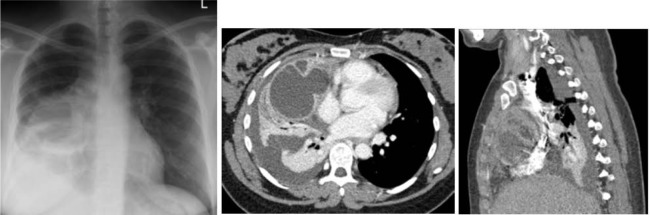
1) chest x-ray showing large mass in the right chest. 2) CT scan with cystic tumor in the anterior mediastinum displacing heart and lung

### Cardiovascular Implantable Electronic Device explantation: What happens after device removal? Perioperative management of pocket infections.

(Abstract ID: 348)

M. Sindt^1^

^1^*Universitätsklinikum Dresden*

**Background:**

The incidence of Cardiovascular Implantable Electronic Device (CIED) pocket infections is continuously on the rise in recent decades. Since the dawn of new extraction methods most papers solely concentrate on the extraction aspect of CIED infections. Our goal was to focus on the postoperative course, wound management and antibiotic therapy.

**Materials and methods:**

All patients that underwent device extraction with pocket infection between Jan 2012 and Dec 2016 were included in this study and were evaluated retrospectively. This includes device infections with lead vegetations. Patients who underwent device extraction without concomitant pocket infection were excluded.

**Results:**

157 patients (76% male, mean age 73±12 years) underwent CIED explantation. The most common complication was postoperative bleeding requiring additional surgery (5%). Our initial approach was to insert a vacuum dressing after extraction. However, we observed increased postoperative bleeding. Afterwards we changed our strategy by inserting Betaisodona® gauze pads. While there was a total reduction of postoperative bleeding (2 incidents with the latter approach, 6 with the former), there was no statistically significance. Other complications include cardiac tamponade (1,2%) and sepsis (1,9%). The total hospital mortality was 3,8% with 6 deaths. Sole pocket infections had a mortality of 1,45%. With the addition of lead vegetations the mortality increased significantly to 27% (3 out of 11 patients, p<0.0001). Higher preoperative CRP was a statistically significant predictor for hospital mortality (p<0.0085). Patients with St. aureus infections had a statistically significant higher CRP than other pathogens (p<0.0001 to p<0.0015) except for P. aeruginosa and MRSA. 21% of patients did not receive a new device. Half of the patients did not have a positive wound swab. The detected pathogens were resistant against the initial antibiotic treatment in 29% of the cases. There was no statistical significance between the initial pathogen and secondary wound closure as well as re-implantation of a CIED (with the exception of MRSA compared to sterile wound swabs, 3 cases of MRSA, p<0,048).

**Conclusion:**

CRP and lead vegetations are statistically significant predictors for intrahospital mortality, while localized pocket infections had low overall mortality. Antibiotic therapy in sterile wound swabs without signs of systemic infections should be reconsidered, since it proved ineffective in 29% of the cases.

### With new techniques come new challenges: Lung herniation after lateral thoracotomy

(Abstract ID: 545)

Z. Fajfrova^1^, A. Darwisch^1^, U. Kappert^1^, K. Matschke^1^

^1^*Universitätsklinikum Dresden*

**Background:**

As cardiothoracic surgery evolves and new minimal invasive techniques appear, so does the number of new complications. We report our experience on several cases of incisional intercostal lung herniation after anterolateral thoracotomy. Optimization of the thorax closure and opening strategies considering the risk factors are discussed.

**Materials and methods:**

Approximately 300 patients received minimal invasive aortic valve replacement via lateral thoracotomy at our clinic. Two patients presented weeks after initial hospital discharge with symptomatic incisional intercostal lung herniation (incidence 0.7%). The patients had a body-mass-index that exceeded 28, one of them was a smoker and another suffered from chronic obstructive pulmonary disease. The repair was performed by using an absorbable polyglactin surgical mesh (Vicryl mesh, Ethicon®). The mesh was placed intrathoracally and fixated to the 2nd and 3rd rib using nonresorbable percostal sutures.

**Results:**

All cases were treated successfully. There was no perioperative mortality. The patients were discharged 7 days after the repair surgery. No relapse was observed after a 2 months follow-up.

**Conclusion:**

The incidence of incisional hernias is around 7.5%. A reconstruction of the thoracic wall combined with the implantation of surgical mesh is recommended for the treatment of intercostal lung herniation. Because the incisional hernias are usually a result of inadequate closure of the chest wall, attention is needed by closing and also opening the thoracotomy incision, especially in high risk patients. As a prevention, a primary incision should be made centrally in the intercostal muscle. For approximating the ribs and for contraction of the intercostal space with pericostal stitches due PDS suture may be considered, especially in the presence of risk factors for herniation and also when there is a distance between two ribs more than 1cm.

### Use of incisional negative pressure wound therapy for prevention of wound healing issues after thoracoabdominal aortic repair

(Abstract ID: 865)

P. Dohmen^1^

^1^*Universitäres Herzzentrum Rostock*

**Background:**

Due to the highly invasive nature of thoracoabdominal aorta replacement, the potential risk for wound complications is given resulting from extensive skin incision, prolonged surgical times, intra- and post-operative fluid shifts, as well as patient’s co-morbidity. A new approach was introduced to improve surgical would healing by using negative pressure wound therapy (NPWT).

**Materials and methods:**

Out of fourteen patients treated with thoracoabdominal aortic repair between May 2016 and August 2018 the most recent four patients underwent surgical incision management using PREVENA PLUSTM. These four patients (male (n=2), female(n=2)) treated with aortic repair of Crawford Extension Type I (n=1) or Type II (n=3) with a median age of 64 years (range 51 to 78 years). One patient suffered from Marfan Syndrome. The NPWT was applied directly at the end of the procedure instead of a standard dry wound dressing and was kept in place for approximately seven days using a continuous negative pressure of 125 mm Hg. An additional period of 5-7 days was implemented in two patients.

**Results:**

There was no in-hospital or 30-day mortality in the observed group. All four patients were discharged to our collaborative rehabilitation centers via the ward (n=2) or the intensive care unit (n=2). Permanent control of the integrity of the system and suction was sufficient at all times. No wound healing issues were observed with limited secretion, and absence of wound infection or dehiscence in all patients. Mobilization was facilitated due to a better stabilization of the wound due to NPWT.

**Conclusion:**

Additional negative pressure wound therapy lead to an improvement of outcome regarding any wound complication after thoracolaparotomy and facilitated mobilization and wound dressing management in the initial postoperative phase.

